# Phrase truncation in PubMed searches

**DOI:** 10.5195/jmla.2017.252

**Published:** 2017-10-01

**Authors:** Roberta Shanman

**To the editor,** in their “Methods” section, the authors of “Supplementary Searches of PubMed to Improve Currency of MEDLINE and MEDLINE In-Process Searches via Ovid” state that “PubMed does not accept truncation when phrase searching, so we had to use numerous phrases to capture as many variations as possible.”

**Figure 1:**
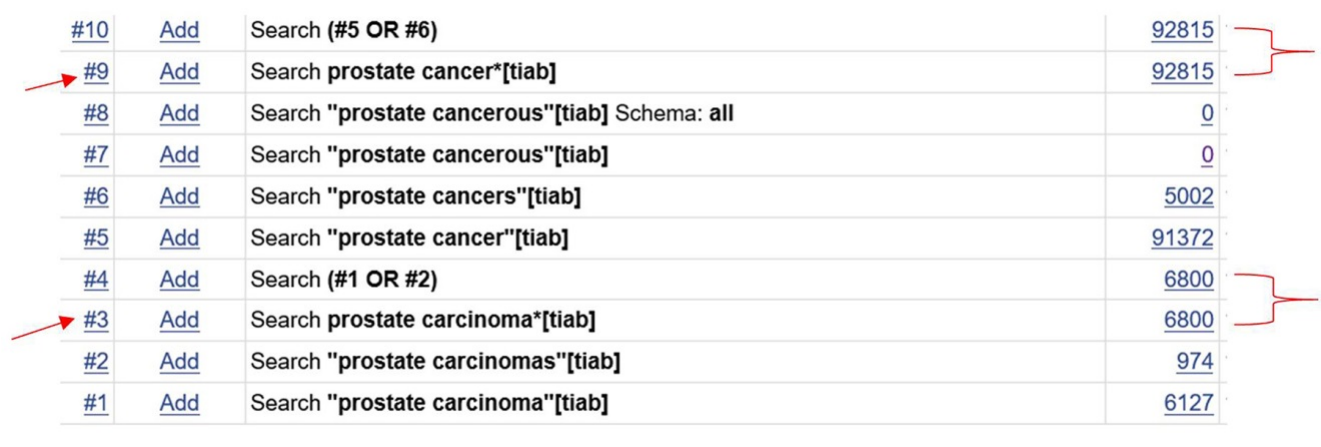
Sample truncation in PubMed

In fact, if an asterisk is added to the end of a phrase, truncation is performed. No double quotes are required around the phrase, as the asterisk acts as a phrase marker as well as truncation. The PubMed searches in the supplementary material duplicate the search string used in the article. Sets 3 and 9 utilize the phrase truncation feature (figure 1). The number of results is the same for the terms using this feature as it is for each individual variation of the term using a Boolean “OR.”

